# Effect of model methanogens on the electrochemical activity, stability, and microbial community structure of *Geobacter* spp. dominated biofilm anodes

**DOI:** 10.1038/s41522-024-00490-z

**Published:** 2024-03-05

**Authors:** Daniel Dzofou Ngoumelah, Tonje Marita Bjerkan Heggeset, Tone Haugen, Snorre Sulheim, Alexander Wentzel, Falk Harnisch, Jörg Kretzschmar

**Affiliations:** 1 DBFZ Deutsches Biomasseforschungszentrum gemeinnützige GmbH (German Biomass Research Centre), Department of Biochemical Conversion, 04347 Leipzig, Germany; 2 SINTEF Industry, Department of Biotechnology and Nanomedicine, 7034 Trondheim, Norway; 3 Helmholtz Centre for Environmental Research - UFZ, Department of Microbial Biotechnology, 04318 Leipzig, Germany; 4 Zittau/Görlitz University of Applied Sciences, Faculty of Natural and Environmental Sciences, 02763 Zittau, Germany

**Keywords:** Applied microbiology, Next-generation sequencing

## Abstract

Combining anaerobic digestion (AD) and microbial electrochemical technologies (MET) in AD-MET holds great potential. Methanogens have been identified as one cause of decreased electrochemical activity and deterioration of *Geobacter* spp. biofilm anodes. A better understanding of the different interactions between methanogenic genera/species and *Geobacter* spp. biofilms is needed to shed light on the observed reduction in electrochemical activity and stability of *Geobacter* spp. dominated biofilms as well as observed changes in microbial communities of AD-MET. Here, we have analyzed electrochemical parameters and changes in the microbial community of *Geobacter* spp. biofilm anodes when exposed to three representative methanogens with different metabolic pathways, i.e., *Methanosarcina barkeri*, *Methanobacterium formicicum*, and *Methanothrix soehngenii*. *M. barkeri* negatively affected the performance and stability of *Geobacter* spp. biofilm anodes only in the initial batches. In contrast, *M. formicicum* did not affect the stability of *Geobacter* spp. biofilm anodes but caused a decrease in maximum current density of ~37%. *M. soehngenii* induced a coloration change of *Geobacter* spp. biofilm anodes and a decrease in the total transferred charge by ~40%. Characterization of biofilm samples after each experiment by 16S rRNA metabarcoding, whole metagenome nanopore sequencing, and shotgun sequencing showed a higher relative abundance of *Geobacter* spp. after exposure to *M. barkeri* as opposed to *M. formicicum* or *M. soehngenii*, despite the massive biofilm dispersal observed during initial exposure to *M. barkeri*.

## Introduction

*Geobacter* spp. are among the most studied electroactive microorganisms (EAM) and are model organisms for studying primary microbial electrochemical technologies (MET)^1,2^. *Geobacter* spp. form multilayer biofilm anodes that can produce current densities of more than 1.0 mA cm^−2^ ^3,4^. Acetate is the preferred carbon source and electron donor of *Geobacter* spp^5^, however they are able to utilize also other volatile fatty acids (VFA) such as lactate and formate as well as hydrogen^6,7^. Electrons derived from the oxidation of VFA are delivered to dissolved or solid terminal electron acceptors (TEA), for the latter by means of extracellular electron transfer (EET)^4,5,7^. TEA are, e.g., elemental sulfur, iron(III) pyrophosphate or electrodes poised at potentials between ~ 0 and 0.4 V vs. standard hydrogen electrode (SHE)^8^. Generally, EET occurs in two different ways: 1) via primary mediators (e.g., H_2_, formate) or secondary mediators (e.g., flavins, phenazine derivates, quinones), known as mediated or indirect EET^9^, or 2) via cytochrome c and nanowires (pili), being called direct EET^5,9^. *Geobacter* spp. are the model for direct EET and have been extensively studied over the last decades, also for potential large-scale MET applications. Among these, microbial fuel cells (MFC) are at the forefront. MFC allow the exploitation of the chemical energy stored in liquid organic material, especially wastewater, to generate electric energy, and are thus being considered a sustainable alternative for wastewater treatment^10–12^, e.g., for bioremediation of nitrate-polluted groundwater^13^. Microbial electrolysis cells (MEC)^14,15^ and the associated microbial desalination cells (MDC)^12,16^ can be used for the production of H_2_ and CH_4_^10,14,15,17^, and the desalination of brackish water and urine^12,16^, respectively. Engineering and implementation of all MET systems still faces several limitations and they are therefore not yet widely commerciallized^18^.

Methanogenic archaea are strictly anaerobic microorganisms, widely involved in anaerobic digestion (AD) for CH_4_ production from biomass^19,20^. Methanogenesis represents the final out of four microbiological conversion steps during AD, after hydrolysis, acidogenesis, and acetogenesis^21,22^. In AD, methanogens live in a syntrophic relationship with fermenting bacteria that break down complex organic substances into mainly acetate, CO_2_, and H_2_^22,23^. Subsequently, biogas (i.e., a mixture of mainly CH_4_ and CO_2_) is produced by methanogens either autotrophically by metabolizing CO_2_ and H_2_ (hydrogenotrophic methanogenesis), or heterotrophically by metabolizing formate, acetate, and short-chain methylated compounds like e.g., methanol (acetoclastic and methylotrophic methanogenesis)^23–27^. The biogas can subsequently be separated from CO_2_ to gain pure CH_4_ that can be injected into the gas grid^21^.

MET in combination with AD, denoted MET-AD, has gained wide scientific interest in recent years, as it aims to increase the efficiency and diversity of AD technologies^28^. Special attention has been given to *Geobacter* spp. dominated biofilm anodes (for simplicity referred to as *Geobacter* spp. biofilms), used e.g., as biosensor receptors for highly time resolved monitoring of VFA in AD^5,29^. Furthermore, the removal of recalcitrant pollutants^30^ and the simultaneous lowering of chemical oxygen demand (COD)^31^ from digestate (i.e., effluents from AD reactors), by means of *Geobacter* spp. biofilms broaden the application spectrum of MET-AD. However, to meet these applications, a long lifespan/high stability and constant electrochemical activity of *Geobacter* spp. biofilms in complex AD environments are required. Recent studies have shown controversial effects of AD effluents on the performance of pre-grown *Geobacter* spp. biofilms^29,32,33^. Some studies reported a massive biofilm dispersal, a reduction in electrochemical activity as well as a decrease in the relative abundance of *Geobacter* spp. biofilm upon exposure to AD effluents^29,32^, while other studies reported the opposite^33^. These studies imply that the (microbial) composition of the AD-effluent makes a difference in terms of reducing the electrochemical activity and relative abundance of *Geobacter* spp. in biofilms. Among the possible causes for this controversial biofilm behaviour in AD effluents are, e.g., high ammonium concentration ( ≥ 4 g L^−^^1^)^29^, protozoans^34^, soluble terminal electron acceptors such as humic substances, nitrate or Iron(III) salts^11,35–37^, and methanogenic archaea which are suspected to play a key role^29,32^. Previous work revealed a wide diversity of methanogens in AD reactors operated under mesophilic conditions, mainly dominated by acetoclastic (e.g., *Methanosarcinaceae* and *Methanosaetaceae*) and hydrogenotrophic (e.g., *Methanobacteriaceae*) methanogenic families^20^. Some methanogens (e.g., *Methanosarcinaceae* and *Methanosaetaceae*) have been reported to be able to accept electrons from *Geobacter* spp. for CH_4_ production, and therefore, may immediately interact with *Geobacter* spp. biofilms^37–39^. Here, electron transfer proceeds mainly in two different ways: 1) direct interspecies electron transfer (DIET) using conductive pili and outer membrane cytochrome c^37,38,40–42^, and 2) mediated interspecies electrons transfer (MIET) also known as H_2_ interspecies transfer (HIT)^25,41–43^. To date, the interactions between *Geobacter* spp. biofilms and methanogens in complex AD-MET systems remain poorly explored. Therefore, taking advantage of MET-AD combinations would require understanding the different interactions between *Geobacter* spp. biofilms and the methanogens occurring in AD which, in some cases, might induce a reduction in electrochemical activity, stability and relative abundance of *Geobacter* spp. in electroactive biofilms^32^.

The objective of this study was to examine the effect of model methanogens, i.e. *Methanosarcina barkeri* (mixotrophic methanogen), *Methanobacterium formicicum* (hydrogenotrophic methanogen), and *Methanothrix soehngenii* (acetotrophic methanogen), on the electrochemical activity, stability, and microbial community structure of pre-grown *Geobacter* spp. biofilm anodes. To achieve this goal, the electrochemical activity and stability of pre-grown *Geobacter* spp. biofilm anodes were monitored during exposure to axenic cultures of each methanogen. To support the electrochemical results, the bacterial and archaeal community of *Geobacter* spp. biofilm samples and selected planktonic samples collected at the end of each experiment were analyzed at the DNA level using 16S rRNA metabarcoding, whole metagenome nanopore sequencing, and whole metagenome shotgun sequencing.

## Results and discussion

### Activity of methanogens in *Geobacter* spp. media

During the biological control experiment, the acetate consumption of *M. barkeri* and *M. soehngenii*, the formate consumption of *M. formicicum* as well as the headspace gas composition were measured (Supplementary Fig. 1). The acetate and formate consumption profiles (Supplementary Fig. 1a), as well as the CH_4_ production (Supplementary Fig. 1b) provide evidence that all three methanogenic archaea remained active in their respective media. Therefore, it was concluded that also mixing 50:50, v/v of acetate-based medium with methanogenic cultures grown in BFS01 medium to study specific interactions with *Geobacter* spp. biofilms in the single chamber MEC is not detrimental to methanogens. For a more detailed discussion of the results, please refer to the supplementary results, section 2.

### Electrochemical performance of *Geobacter* spp. biofilms in a 50:50, v/v mixture of acetate-based medium and abiotic BFS01 medium

**Fig. 1:**
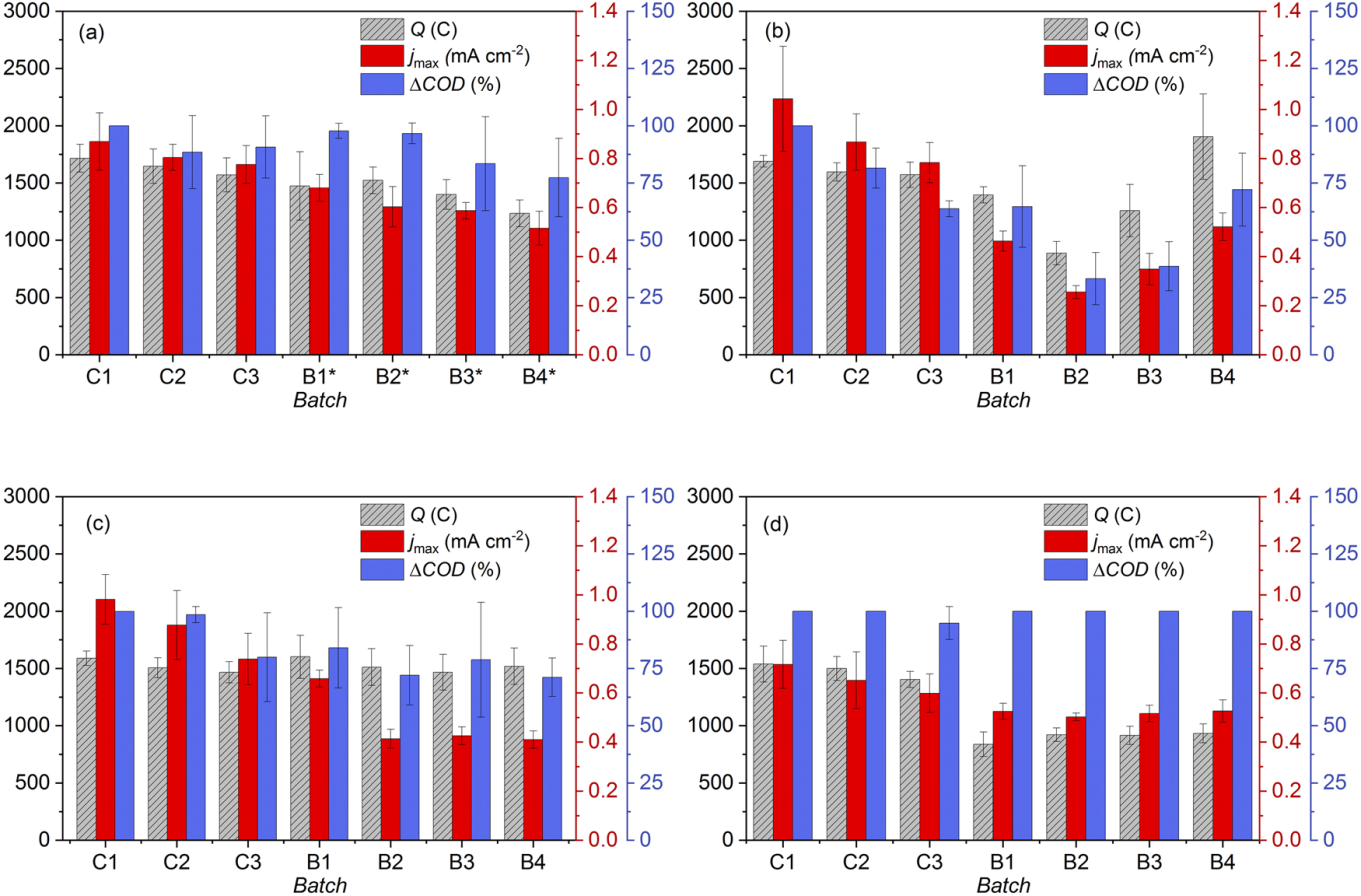
*Geobacter* spp. biofilms exposed to varied media conditions. Transferred charge (${\rm{Q}}$), current density (*j*_max_) and chemical oxygen demand removal (ΔCOD) upon exposure of *Geobacter* spp. biofilms to a 50:50, v/v mixture of acetate-based medium and: (**a**) abiotic BFS01 medium, (**b**) *M. barkeri* cultures in BFS01, (**c**) *M. formicicum* cultures in BFS01, (**d**) *M. soehngenii* cultures in BFS01. C1-C3: successive control batches with only acetate-based medium, B1*-B4*: four successive batch cycles with abiotic BFS01 medium, B1-B4: four successive batch cycles with the methanogenic cultures grown in the BFS01 medium (see Fig. 4), *n* ≥ 3, error bars indicate confidence interval CI.

The electrochemical activity and stability of *Geobacter* spp. biofilms in the 50:50, v/v mixture of acetate-based medium and BFS01 medium were monitored (Fig. 1a). The control batches using only acetate-based medium (C1-C3, see Fig. 4) already showed a slight decrease in the mean values of transferred charge ($Q$) and maximum current density (*j*_max_) over time. This is related to the natural maturation of *Geobacter* spp. biofilms^44^. A similar slight decrease in the mean values of $Q$ and *j*_max_ was also observed during exposure to BFS01 medium without methanogens (B1* to B4*, see Fig. 4) due to ongoing maturation. Acetate utilization by *Geobacter* spp. biofilm and other microorganisms embedded in the biofilm structure decreased by ~23% from C1 to B4*, as also shown by the ΔCOD. Comparison of successive control batch cycles and exposure batch cycles (C1-C3 and B1*-B4*) revealed an insignificant decrease of all parameters studied (i.e., $Q$, *j*_max_, and ΔCOD), as indicated by overlapping confidence interval (CI) in Fig. 1a. This is consistent with the coulombic efficiency (*CE*) values which show no significant variations from C1 to B4* (Supplementary Table 2, Electrochemical control).

In mixed species biofilms, *Geobacter* spp. have been found to inhabit the inner biofilms near the electrode surface, while other microorganisms grow on the outer layers^45^. This indicates that with maturation, more non-electroactive microorganisms form the outer layers of the biofilm where they consume substrates and reduce diffusion of substrates into the inner layers where they are oxidized by EAM. This results in a slight decrease in electrochemical biofilm performance, as observed from C1 to B4*. Therefore, we conclude that the 50:50, v/v mixture of acetate-based medium and BFS01 medium used from B1* to B4*, has no negative effect on the performance of *Geobacter* spp. biofilms.

### Electrochemical activity and stability of *Geobacter* spp. biofilm upon exposure to *M. barkeri*

The electrochemical activity and stability of *Geobacter* spp. biofilms in the 50:50, v/v mixture of acetate-based medium in presence of *M. barkeri* were monitored (Fig. 1b). It was observed that the mean values of $Q$ and *j*_max_ gradually but insignificantly decreased with increasing number of control batches (C1 to C3), while ΔCOD already showed a significant decrease during the control batches. The latter observations can be directly linked to the maturation of the biofilm leading to the formation of non-electroactive microorganisms in the outer layers of the biofilm that could decrease acetate diffusion^44^. Compared to the last control batch (C3), the first exposure batch (B1), showed no significant decrease in the mean values of $Q$ and ΔCOD, as indicated by overlapping CI. This is in contrast to the mean value of *j*_max_ which dropped by ~ 41%. From the first to the second exposure batch (B1 to B2), the mean values of $Q$, *j*_max_, and ΔCOD significantly decreased. Unexpectedly, biofilm performance reversed in the third and fourth exposure batch (B3 & B4), as indicated by a significant increase in the mean values of $Q$ and *j*_max_. An increase was also observed in the mean values of ΔCOD, but this was only significant from B3 to B4. Comparison of the last control batch (C3) with the last exposure batch (B4), indicated no significant difference in the mean values of $Q$ and ΔCOD, while *j*_max_ values remained slightly lower in B4 than in C3.

**Fig. 2:**
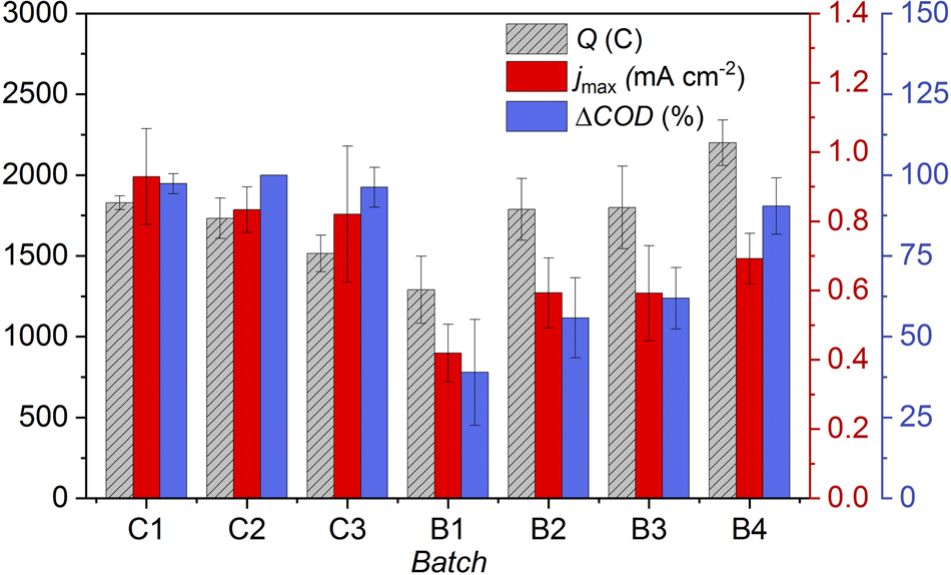
Exploring the effect of *M. barkeri* age on *Geobacter* spp. biofilm. Transferred charge (${\rm{Q}}$), current density (*j*_max_) and chemical oxygen demand removal (ΔCOD) at the end of each batch cycle upon exposure of *Geobacter* spp. biofilm to a 50:50, v/v mixture of acetate-based medium and *M. barkeri* cultures in BFS01 medium. C1-C3: control batches with only acetate-based medium, B1-B4: four successive batch cycles with *Geobacter* spp. biofilms exposed to *M. barkeri* cultures aged 3–6 weeks, respectively, *n* = 4, error bars indicate confidence interval CI.

To better understand the increase in biofilm performance observed from B3 when consistently using 3-week-old *M. barkeri* cultures from B1 to B4 (Fig. 1b), a similar experiment was conducted using *M. barkeri* cultures aged 3 to 6 weeks from B1 to B4, respectively (Fig. 2). It was observed that the previous decrease in biofilm performance in B2 (Fig. 1b), already occurred in B1 for *j*_max_ and ΔCOD. However, subsequent batches showed an increase in $Q$, *j*_max_, and ΔCOD, with values in the last exposure batch B4 overlapping (*j*_max_ and ΔCOD) or exceeding ($Q$) those in the last control batch C3 (Fig. 2). Thus, it seems that the age of the *M. barkeri* cultures used here (i.e., 3-week-old or older) had no direct influence on the interactions with *Geobacter* spp. biofilms.

The results in Figs. 1b and 2 show that *Geobacter* spp. biofilms were significantly affected by *M. barkeri* during the first two exposure batches. The observed decrease in the performance of *Geobacter* spp. biofilms was evidenced by a visually perceptible dispersal (Supplementary Fig. 3b), showing the detachment of the outer biofilm layers. Visual examination of the biofilm structure in successive batches (B3 to B4) also indicated dispersal of outer biofilm layers, coupled to the formation of new biofilm layers underneath the dispersing layers (data not shown). This is in line with studies suggesting that *Geobacter* spp. dominate in the near-electrode portion of the biofilm^45^. The formation of new biofilm layers during biofilm dispersal may indicate that *M. barkeri* mainly affected the outer layers of anodic biofilms, allowing acetate to easily reach the inner layers resulting in increased *Geobacter* spp. activity. The latter assumption is supported by the mean *CE* values (Supplementary Table 2, M*. barkeri* + *Geobacter* spp. biofilms), which increased significantly in the batch cycles showing stronger biofilm dispersal (i.e., B2 in Fig. 1b and B1 in Fig. 2). The obtained overestimated *CE* values (*CE* > 100%) may also have been induced by H_2_ produced at the cathode that can be consumed by *Geobacter* spp^46,47^. as well as *M. barkeri* and other microorganisms that may cross-feed *Geobacter* spp^15,48^. In the batch cycles following stronger dispersal of the outer biofilm layers (i.e., from B3 in Fig. 1b and B2 in Fig. 2), the mean *CE* values remained nearly constant or were slightly decreased, respectively. This is consistent with the work of, e.g., Korth et al. ^47^, who reported that newly formed *Geobacter* spp. biofilms utilize a large proportion of electrons from acetate oxidation for biomass production during growth, mainly resulting in decreased *CE*.

For a better understanding of the previously described variations in the electrochemical activity and stability of *Geobacter* spp. biofilm when exposed to *M. barkeri*, it is also important to consider the metabolic properties of *M. barkeri*. Compared to enriched biofilms of *Geobacter* spp. in single chamber MEC batch reactor, *M. barkeri* cultured at 37 °C is reported to have a lower affinity for acetate (Michaelis-Menten constant, K_M_ of 0.67 mM for *Geobacter* spp. vs. 5 mM for *M. barkeri*)^4,49,50^. Therefore, we assume that the newly formed biofilm layers by *Geobacter* spp. may have outcompeted *M. barkeri* in substrate utilization under the experimental conditions used, resulting in the observed increase in biofilm performance from B3 in Fig. 1b and B2 in Fig. 2.

Members of the *Methanosarcinaceae* are reported to be able to establish DIET with EAM^27,38^. It has been proposed that DIET between *Methanosarcinaceae* and EAM is facilitated by multiheme cytochrome c. Yet, cytochromes are not essential for DIET, as some species, e.g., *M. barkeri*, lacking multiheme complexes can still perform DIET^27,38^. Referring to Fig. 1b and 2, it can be deduced that, during B1 and B2, DIET may have occurred, resulting in biofilm dispersal. This indicates that DIET-related genes are likely expressed in the biofilm outer layers that mainly dispersed in B1 and B2. Therefore, we strongly advocate follow-up studies shedding light on the expression of DIET-related genes in the *Geobacter* spp. biofilm structure. Furthermore, advanced techniques such as scanning electron microscopy and/or optical coherence tomography (which enables direct investigation of biofilms inside the cultivation device) would be very useful in future studies for in-depth investigation of changes in biofilm morphology^51,52^.

### Electrochemical activity and stability of *Geobacter* spp. biofilm upon exposure to *M. formicicum*

The electrochemical activity and stability of *Geobacter* spp. biofilms in the 50:50, v/v mixture of acetate-based medium and *M. formicicum* cultures were monitored (Fig. 1c). It was observed that the mean values of $Q$, *j*_max_, and ΔCOD decreased gradually but insignificantly with increasing number of control batches (C1 to C3). This is in line with the observation for *Geobacter* spp. biofilms in a 50:50, v/v mixture of acetate-based medium and abiotic BFS01 (Fig. 1a). Between the last control batch (C3) and the subsequent first exposure batch (B1), no significant differences were observed for all monitored parameters, as indicated by overlapping CI. Compared to B1, the mean value of *j*_max_ in the second exposure batch (B2) dropped significantly by ~ 37% and remained nearly constant until the last exposure batch (B4). In contrast to *j*_max_, the mean values of $Q$ and ΔCOD remained nearly constant from B1 to B4 and overlapped with the last control batch (C3). In general, the mean values of $Q$ and ΔCOD upon biofilm exposure to *M. formicicum* (Fig. 1c) showed a similar trend to the control experiment without methanogens (Fig. 1a). This was evidenced by a gradual but insignificant decrease of both parameters in successive control and exposure batch cycles (C1 to B4). Similarly, *CE* values did not vary significantly when comparing C3 and each exposure batch cycle (Supplementary Table 2, M*. formicicum + Geobacter* spp. biofilms), which is indicating a limited effect of *M. formicicum* on the performance of *Geobacter* spp. biofilms.

*M. formicicum* is known as a hydrogenotrophic methanogen that uses H_2_ and formate as electron source for the reduction of CO_2_ to CH_4_, following the HIT pathway^41,53–55^. H_2_ can be produced in MEC via the reduction of protons at the cathode, and then used as an electron donor by *M. formicicum* and *Geobacter* spp^47,56^. However, *M. formicicum* is known to have a high growth rate and doubling time of only a few hours under H_2_-CO_2_ feeding^53,57^. Therefore, we hypothesize that *M. formicicum* cells consumed a large share of the H_2_ produced at the cathode. Putative H_2_ consumption by *M. formicicum* may explain the decrease in *j*_max_ observed in B2 (Fig. 1c), although *CE* did not vary significantly from B1 to B2 (Supplementary Table 2). Similar biofilm performance was observed by Korth et al. ^47^ who estimated the contribution of H_2_ to biofilm performance, using a two-chamber MEC with and without additional H_2_ as electron donor in the anode compartment. Whereas *j*_max_ in B1 was nearly identical to *j*_max_ in C3, suggesting that *M. formicicum* needed time to adapt to the growth conditions, $Q$ and ΔCOD were not significantly affected in any of the batches of *Geobacter* spp. biofilm exposure to *M. formicicum* due to the inability of the latter to metabolize acetate. Furthermore, *Methanobacteriaceae* (e.g., *M. formicicum*) have been shown to be unable to perform DIET, meaning that they cannot accept electrons from acetate oxidation by *Geobacter* spp. to reduce CO_2_ to CH_4_^41,58^.

Visual examination of *Geobacter* spp. biofilm after exposure to *M. formicicum* indicated no or limited biofilm dispersal to the planktonic phase (Supplementary Fig. 3c).

### Electrochemical activity and stability of *Geobacter* spp. biofilm upon exposure to *M. soehngenii*

The electrochemical activity and stability of *Geobacter* spp. biofilms in the 50:50, v/v mixture of acetate-based medium and *M. soehngenii* cultures were monitored (Fig. 1d). It was observed that the mean values of $Q$ and *j*_max_ decreased slightly but insignificantly with increasing number of control batches (C1 to C3). This is also in line with $Q$ and *j*_max_ for *Geobacter* spp. biofilms in the 50:50, v/v mixture of acetate-based medium and abiotic BFS01 (Fig. 1a). A minor but insignificant decrease in ΔCOD was observed only in C3. Compared to the last control batch (C3), the mean value of $Q$ in the first exposure batch to *M. soehngenii* (B1) dropped by ~40%, unlike the mean value of *j*_max_ which decreased insignificantly. From B1 to the last exposure batch (B4), both $Q$ and *j*_max_ remained nearly constant, while COD was always completely removed. The *CE* also dropped by ~34% from C3 to B1 and afterwards remained nearly constant (see Supplementary Table 2, M*. soehngenii + Geobacter* spp. biofilms).

*M. soehngenii* is an obligate acetoclastic methanogen^38,59–61^. Like other members of the *Methanosaetaceae*, *M. soehngenii* lacks genes involved in the previously described HIT pathway^40^. Although lacking multiheme cytochrome c, certain members of the *Methanosaetaceae*, such as *M. soehngenii* have been reported to perform DIET, as they can accept electrons from acetate/ethanol oxidation by *Geobacter* spp. (e.g., *G. metallireducens/G. sulfurreducens*), for the reduction of CO_2_ to CH_4_^38,40,41^. However, the mean value of *j*_max_ in the last exposure batch (B4) (Fig. 1d) is nearly identical to the mean value of *j*_max_ in the last batch of the control experiment without methanogens (B4) (Fig. 1a). Therefore, we speculate that DIET between *Geobacter* spp. biofilm and *M. soehngenii* does not occur or occurs only at a limited rate and does not induce significant dispersal of the biofilm outer layers.

Chronoamperograms obtained upon exposure of *Geobacter* spp. biofilms to *M. soehngenii* showed no current production from day 4 to 5 of each batch cycle (B1-B4), indicating acetate limitation (Supplementary Fig. 4). The acetate limitation for *Geobacter* spp. biofilm during the last 2–3 days of each batch cycle, may also explain the significant drop in the mean value of $Q$ (Fig. 1d) and CE (Supplementary Table 2) observed from C3 to B1.

After exposure to *M. soehngenii*, outer layers of *Geobacter* spp. biofilms appeared dark brownish in contrast to the inner biofilm layers which appeared reddish (Supplementary Fig. 3d), almost similar to the control biofilm (Supplementary Fig. 3a). *M. soehngenii* is well known for its high affinity for acetate (K_M_ of 0.4–0.8 mM)^60,62,63^, which is similar to that of *Geobacter* spp. (K_M_ of 0.67 mM)^4^, and can consume acetate at very low concentrations (7–70 µmol L^−^^1^)^64^ making it a strong competitor for acetate. The competition for acetate and the high affinity of *M. soehngenii* for acetate may have led to severe starvation of *Geobacter* spp., especially in the outer biofilm layers, during the last 2-3 days of each batch cycle from B1 to B4. Following the latter hypothesis, we speculate that cytochromes in the outer membranes of *Geobacter* spp. cells were oxidized, which could lead to the observed change in biofilm color. However, as *M. soehngenii* does not metabolize H_2_, we assume that *j*_max_ values observed from B1 to B4 are the contribution of electrons from acetate oxidation by *Geobacter* spp. biofilm and part of the H_2_ produced at the cathode. Why the outer layers of the *Geobacter* spp. biofilm did not disperse in the presence of *M. soehngenii* but in the presence of *M. barkeri*, albeit both species can perform DIET, cannot be answered by our data. Answering this question would require, e.g., a much longer exposure of *Geobacter* spp. biofilm to both methanogens, a change ratio of the mixture acetate-based medium and methanogens, or knock out/down mutants of specific DIET related genes.

### Molecular biology analysis

To trace possible changes in the microbial community of *Geobacter* spp. biofilms and to relate those changes to the electrochemical biofilm performance (i.e., $Q$, *j*_max_, ΔCOD and CE), three biological replicates of biofilm samples at the end of each experiment and selected planktonic samples were analyzed by 16S rRNA metabarcoding, whole metagenome shotgun sequencing, and whole metagenome nanopore sequencing for subsequently comparison. Control biofilms (i.e., biofilms after exposure to the 50:50, v/v mixture of acetate-based medium and abiotic BFS01 medium) were used as a benchmark. Biofilm samples were analyzed with emphasis on the relative abundance of *Geobacter* spp. and methanogenic communities in the biofilms.

**Fig. 3:**
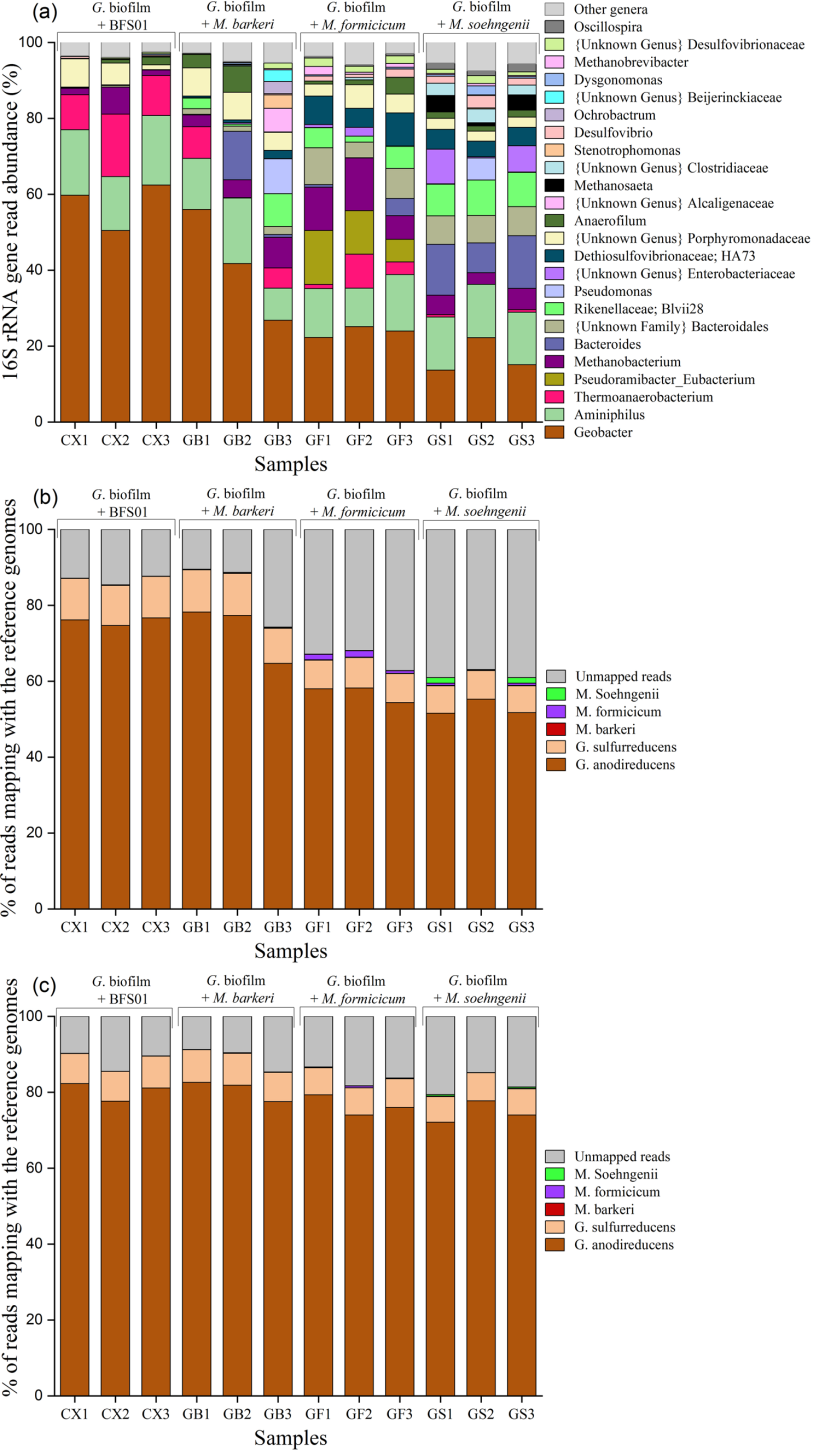
Characterisation of *Geobacter* spp. biofilm using varied profiling techniques. **a** Metabarcoding abundance profiling targeting the 16S rRNA V3-V4 region, (**b**) whole metagenome shotgun read percentages, and (**c**) whole metagenome nanopore read percentages of biofilm samples at the end of the experiment upon exposure of *Geobacter* spp. biofilm to the abiotic BFS01 medium and each methanogenic culture in BFS01 medium, respectively. [CX1-CX3], [GB1-GB3], [GF1-GF3], and [GS1-GS3] indicate triplicate biological biofilm samples after each electrochemical experiment, i.e., CX1-CX3: after biofilm exposure to abiotic BFS01 medium, GB1-GB3: after biofilm exposure to *M. barkeri*, GF1-GF3: after biofilm exposure to *M. formicicum*, and GS1-GS3: after biofilm exposure to *M. soehngenii*, respectively. (Other genera < 2%).

The 16S rRNA metabarcoding analysis at the genus level indicated a relative abundance of *Geobacter* spp. of ~58% in control biofilms, ~42% after exposure to *M. barkeri*, ~24% after exposure to *M. formicicum*, and ~17% after exposure to *M. soehngenii* (Fig. 3a). Other bacteria such as the genus *Aminiphilus* and the family *Porphyromonadaceae* occurred inconsistently in all biofilm samples. In contrast, the genus *Bacteroides*, not detected in control biofilms, showed a relative abundance of ~5%, ~2%, and ~12%, in biofilms after exposure to *M. barkeri*, *M. formicicum* and *M. soehngenii*, respectively. The genus *Thermoanaerobacterium* with a relative abundance of ~12% in control biofilms, decreased to ~5% in biofilms after exposure to *M. barkeri* and *M. formicicum*, and was barely detected in biofilms after exposure to *M. soehngenii*. The metabarcoding analysis showed that the main dominant methanogenic community in all biofilm samples was the genus *Methanobacterium* with a relative abundance of ~3% in control biofilms, ~11% in biofilms after exposure to *M. formicicum*, and ~5% in biofilms after exposure to *M. barkeri* and *M. soehngenii*. The methanogenic community in *Geobacter* spp. dominated biofilms grown in acetate-based medium is reported to be ~90% dominated by the genus *Methanobacterium*^47^. This may explain the overall relative abundance of ~3% *Methanobacterium* spp. detected in the control biofilm metagenome. Overall, it seems that, in the order of their appearance, *M. barkeri*, *M. formicicum*, and *M. soehngenii* induce a gradual decrease in the relative abundance of *Geobacter* spp. in anodic biofilms.

As previously described, biofilm outer layers of *Geobacter* spp. were mostly dispersed initially (B1-B2 in Figs. 1b, 2, and Supplementary Fig. 3b) upon exposure to *M. barkeri* while new biofilm layers were formed during the following batch cycles leading to a recovery of electrochemical activity (B4 in Fig. 1b). The nearly identical *j*_max_ in the last batches of the control experiment (B4* in Fig. 1a) and of the biofilm exposure to *M. barkeri* (B4 in Fig. 1b) is consistent with the similar relative abundance of *Geobacter* spp. observed in both experiments (Fig. 3a). The similar *j*_max_ observed in B4* and B4 also indicate a comparable rate of acetate oxidation and thereby, electrochemical activity of *Geobacter* spp. in these two batches. Furthermore, similar relative abundance of *Geobacter* spp. was observed in the biofilms after exposure to *M. barkeri* aged 3 to 6 weeks from B1 to B4 (Supplementary Fig. 5) and in the biofilms after exposure to *M. barkeri* aged 3 weeks over all exposure batches. This confirms the hypothesis that the age of the *M. barkeri* cultures used here (i.e., 3-week-old or older) had no direct influence on the interactions with *Geobacter* spp. biofilms

Metabarcoding of the planktonic phase in B4 after biofilm exposure to *M. barkeri* indicated a relative abundance of ~16% of *Methanosarcina* spp. and ~11% of *Geobacter* spp. (Supplementary Fig. 6). We assume that the ~11% of *Geobacter* spp. detected in the planktonic phase was caused by biofilm dispersal.

The observed lower relative abundance of *Geobacter* spp. in biofilms after exposure to *M. formicicum* compared to the control biofilms may be related to the variation in the relative abundance of other genera, for example, *Methanobacterium* and *Eubacterium*. *Methanobacterium* spp. are well known to perform HIT with CH_4_ as final product^65^. The relative abundance of *Methanobacterium* spp. in biofilms after exposure to *M. formicicum* was ~3 times higher than in control biofilms indicating an active incorporation of *M. formicicum* in the biofilm outer layers. Therefore, *Geobacter* spp. were most likely limited in supply with cathodic H_2_ due to H_2_ consumption by *M. formicicum*, leading not only to a lower *j*_max_ in B4 (Fig. 1c), but also to a lower relative abundance of *Geobacter* spp. compared to the control biofilms (Fig. 3a).

The relative abundance of the genus *Eubacterium* after biofilm exposure to *M. formicicum* was ~11% in biofilm samples and ~20% in the planktonic phase, unlike in the control biofilm where it was not detected. We assume that exposure of *Geobacter* spp. biofilm to *M. formicicum* enabled growth of *Eubacterium* spp. both in the biofilm and the planktonic phase. Further research on the specific interactions between *Eubacterium* spp., *Geobacter* spp. and *M. formicicum* is needed to shed light on the significant increase in relative abundance of *Eubacterium* spp. and the decrease in relative abundance of *Geobacter* spp. upon exposure to *M. formicicum*.

The relative abundance of *Geobacter* spp. in biofilms after exposure to *M. soehngenii* was ~3 times lower than in the control biofilms (Fig. 3a). This may be directly related to *M. soehngenii* cells in the planktonic phase that were incorporated in the biofilm, which comprised ~3% *Methanothrix* (*Methanosaeta*) spp. versus 0% in the control biofilm. Planktonic samples in B4 after biofilm exposure to *M. soehngenii* were not sequenced due to low DNA recovery. However, the high affinity of *M. soehngenii* to acetate may have caused acetate limitation for *Geobacter* spp. in the biofilms and therefore, triggered the observed decrease in the relative abundance of *Geobacter* spp. Furthermore, the relative abundance of the genus *Thermoanaerobacterium* in control biofilms was ~12% versus 0% in biofilms after exposure to *M. soehngenii*. *Thermoanaerobacterium* spp. have been described as fermenting bacteria involved in the conversion of VFA to CO_2_ and H_2_^66^. Thus, it appears that the high affinity of *M. soehngenii* for acetate (K_M_ of 0.4–0.8 mM)^60,62,63^, not only restricted the utilization of acetate by *Geobacter* spp. (K_M_ of 0.67 mM)^4^ but also hindered or reduced the electrochemical activity of other bacteria. Other microorganisms such as the order *Bacteroidales* (including the genus *Bacteroides*), the families *Rikenellaceae*, *Enterobacteriaceae* and *Dethiosulfovibrionaceae*, which were not detected in the control biofilms, showed significant relative abundance in biofilm after exposure to *M. soehngenii*. However, the effect (positive, negative or neutral) of each of the aforementioned microorganism groups on the biofilm performance needs further investigation in future research.

As the metabarcoding only covers the V3-V4 region of the 16S rRNA gene, which does not allow for sufficient differentiation down to the species level, but is limited to the genus or in some cases only the family level, biofilm samples taken after each experiment were analyzed by whole metagenome shotgun sequencing (Fig. 3b). Special attention was paid to sequence reads mapping to reference genomes of *G. anodireducens*, G*. sulfurreducens*, *M. barkeri*, *M. formicicum*, and *M. soehngenii* and the comparison with the relative metabarcoding abundance of the corresponding genera in Fig. 3a. Unmapped reads, i.e., *Geobacter* spp. sequences not found in the genomes used as references (given that the *Geobacter* spp. biofilm was from a mixed origin and not based on a pure strain) and other organisms, were not analyzed due to the unknown nature of their functional involvement in biofilm performance. *G. anodireducens* and *G. sulfurreducens* are known as two of the main electroactive species of the genus *Geobacter*^45^ forming electroactive biofilms. The raw data mapping showed that ~76% and ~11% of the control sample reads mapped against *G. anodireducens* and *G. sulfurreducens*, respectively. The relative mapping abundance of both species observed in the biofilms after exposure to *M. formicicum* and *M. soehngenii* were reduced to ~57–53% and ~7.8–7.3%, respectively. In contrast the relative abundance of *Geobacter* spp. did not significantly change upon exposure to *M. barkeri*, assumingly due to the observed biofilm recovery after dispersal (vide supra, B3-B4 in Fig. 1b). However, the combined read percentages of the two *Geobacter* spp. in Fig. 3b indicates a clear decrease in the relative abundance of *Geobacter* spp. in all biofilm samples for both methods, i.e., metabarcoding (Fig. 3a) and whole metagenome shotgun sequencing (Fig. 3b). The lower relative abundance measured by metabarcoding compared to shotgun sequencing may be explained by different priming efficiency of the primers used to target archaea and bacteria in the PCR amplification process^67^. In addition, it is worth noting that the copy number of the 16S rRNA gene shows a high variation, where 1–15 copies have been reported in bacterial genomes, while it is typically lower in archaea with 1–4 copies^68,69^. This might, however, not be a major contributor in the current study since it was found that the copy number in the published reference genomes were 2 for *M. soehngenii* (NC_015416), *M. formicicum* (NZ_CP006933), and *G. sulfurreducens* (NZ_CP078092.1), while *M. barkeri* (NZ_CP009528) had 3 copies. No closed genome has been published for any verified *G. anodireducens* and it likely encodes more than the single copy covered by the published draft genome (NZ_JADBFD000000000). Thus, except for *M. barkeri*, which will be over-estimated based on 16S compared to shotgun sequencing, the other organisms (possibly with the exception of *G. anodireducens*) likely show a representative coverage in the amplicon sequencing study. Meanwhile abundance estimates based on read mapping percentages will also be affected by differences in genome size, where larger genomes like those of *M. barkeri* (4.5 Mbp), *G. sulfurreducens* (3.8 Mbp), and *G. anodireducens* (3.7 Mbp) will be overestimated compared to the smaller ones of *M. formicicum* (2.4 Mbp) and *M. soehngenii* (3.0 Mbp), given that the cell lysis otherwise has been complete for all species during DNA extraction. Thus, if the aim were to present a “cell-count” representation of abundance rather than visualizing how the biofilm microbiota composition changed over time, more normalization steps would have had to be included.

Mapping of sequence reads to the reference genomes of *M. barkeri*, *M. formicicum*, and *M. soehngenii* indicated that none of them were present in the control biofilms. Surprisingly, no sequence reads corresponding to the reference genome of *M. barkeri* were identified for biofilms after exposure to *M. barkeri*. This may indicate that *M. barkeri* cells did not integrate into the biofilm structure, which could be one of the reasons why the electrochemical activity of *Geobacter* spp. biofilm could recover after the dispersal of the biofilm outer layers (e.g., B3-B4 in Fig. 1b). Read percentages of ~1.3% mapping to the reference genome of *M. formicicum* was identified for biofilms after exposure to *M. formicicum*. This indicates that *M. formicicum* incorporated into the biofilm structure without affecting its integrity. Furthermore, since *M. formicicum* uses exclusively H_2_ as energy source, we can reinforce our previous hypothesis that the inner layers of *Geobacter* spp. biofilms were deprived of H_2_, which may explain the observed decrease in *j*_max_ and relative abundance of *Geobacter* spp. Read percentages of ~1% mapping to the reference genome of *M. soehngenii* was identified for biofilms after exposure to *M. soehngenii*. *M. soehngenii* is well known to have high affinity for acetate (K_M_ of 0.4–0.8 mM)^60,62,63^, and to perform DIET^38,40,41^. This may have induced strong competition for acetate with *Geobacter* spp. (K_M_ of 0.67 mM)^4^, explaining the lower relative abundance of *Geobacter* spp. observed upon biofilm exposure to *M. soehngenii* compared to control biofilms. In general, it can be assumed that the higher read percentages not matching any reference genome in biofilms after exposure to *M. formicicum* and *M. soehngenii* compared to control biofilms and biofilms after exposure to *M. barkeri* may be related to the incorporation of *M. formicicum* and *M. soehngenii*, in the biofilm structure, probably allowing other species that already exist in the *Geobacter* spp. biofilms to thrive, e.g., via syntrophic interactions. Furthermore, this increase in unmapped reading could be the result of opportunistic microorganisms (having different effects on biofilm performance, or being totally neutral in terms of interaction) growing periodically in mixed cultures, which may have been promoted by either condition.

In contrast to whole metagenome shotgun sequencing, whole metagenome nanopore sequencing of control biofilms and biofilms after exposure to *M. barkeri* indicated read percentages of ~80% and ~8%, for *G. anodireducens* and *G. sulfurreducens*, respectively (Fig. 3c). The read percentages of both species observed in the biofilms after exposure to *M. formicicum* and *M. soehngenii* were reduced to ~76–74% and ~7.3–7.0%, respectively. All three methanogens were scarcely detected in control biofilms (relative abundances ≤ 0.02%) as well as in biofilms after exposure to each methanogen (relative abundances ≤ 0.05–0.3–0.2% for *M. barkeri*, *M. formicicum* and *M. soehngenii*, respectively). Unmapped reads indicated values of ~12% in control biofilms, ~11% after exposure to *M. barkeri*, ~16% after exposure to *M. formicicum*, and ~18% after exposure to *M. soehngenii*.

Similar to whole metagenome shotgun sequencing, whole metagenome nanopore sequencing results indicate slight incorporation of *M. formicicum* and *M. soehngenii* in the biofilms after exposure (6- and 4-fold incorporation of *M. formicicum* and *M. soehngenii* into biofilms compared to *M. barkeri*). Furthermore, read percentages not matching any reference genome in biofilms after exposure to *M. formicicum* and *M. soehngenii* slightly increase compared to control biofilms and biofilms after exposure to *M. barkeri*. The lower unmapped read observed using nanopore sequencing, as opposed to shotgun sequencing, can be explained by the high probability of sequence similarities, but with distinct differences when studying metagenomes. Thus, distinguishing between sequencing errors and real sequence differences becomes difficult when generating assemblies using nanopore sequencing (single-base accuracy ~ 80-90%). Overall, it seems that no major change occurred in the microbial community of the *Geobacter* spp. biofilm regardless of the experimental conditions. However, the electrochemical results and the results from paired-end amplicon sequencing as well as shotgun sequencing showed significant interactions, e.g., variation in *j*_*max*_ when comparing control biofilms with biofilms after exposure to *M. soehngenii*. This leads to the question of the suitability of using nanopore sequencing alone when studying metagenomes. Shotgun sequencing produce short reads with a high accuracy and thus is not as sensitive to DNA fragmentation prior to library preparation compared to nanopore sequencing, which can produce ultralong sequence reads, but at a lower single base accuracy. The nanopore reads were filtered, removing all reads shorter than 1000 bp, while for the Miseq shotgun data the average read-length was typically in the range of 150–200 bp (data not shown). Thus, it cannot be excluded that the results can be affected by DNA extraction efficiency and associated DNA shearing, which might be different in one sample compared to the next. Although shotgun sequencing results appear to be more consistent with electrochemical results compared to nanopore sequencing results, further research may be needed to shed light on which of the two sequencing techniques is best suited to characterize metagenomes in MET. However, it is worth pointing out that this is no general philosophical discussion but a point of pragmatic application to our research.

## Methods

### General remarks

All microbial experiments were conducted under strictly anoxic conditions. All reported potentials refer to the Ag/AgCl sat. KCl reference electrode ( + 0.197 V vs. SHE). All chemicals were of analytical or biochemical grade. Electrochemical experiments were performed as independent biological replicates with *n* ≥ 3 and results are presented as mean values with corresponding confidence interval (CI) at 95% confidence level^70^.

### Experimental setup for growing axenic methanogen cultures and *Geobacter* spp. dominated biofilms

The experimental setup for culturing each methanogenic strain consisted of 200 mL serum bottles closed with butyl rubber stoppers and aluminium crimp seals (LABSOLUTE, Th. Geyer GmbH). The bottles were prepared, rendered anoxic and sterile according to Dzofou et al. ^71^.

The experimental setup for growing *Geobacter* spp. biofilms and performing electrochemical experiments consisted of a three-electrode arrangement, integrated into 250 mL three-neck round bottom flasks that were used as single-chamber MEC, i.e., no separation of anodic and cathodic compartment. The working and the counter electrodes were made of graphite rods (anode: d = 10 mm, L = 20 mm, A = 7.1 cm^2^, cathode: d = 10 mm, L = 30 mm, A = 10.2 cm^2^_,_ quality CP-2200, CP-Graphitprodukte GmbH). Stainless steel wires (d = 0.5 mm, Goodfellow GmbH) and epoxy glue (Toolcraft, Conrad Electronic SE) were used to fabricate the electrodes as previously described^32^. Stainless steel wires were always isolated using a shrink tube made of modified polyolefin (ABB Ltd). The assembled MEC, with the exception of the reference electrodes (Xylem Analytics, Sensortechnik Meinsberg), were autoclave-sterilized at 121 °C for 20 min. Reference electrodes were sterilized in Beckman solution^72^ for at least 2 h and then rinsed with sterile water before use. The three-neck round bottom flasks were closed with polychloroprene stoppers. To avoid overpressure in the MEC during each batch cycle, hollow needles (with attached 0.2 µm syringe filter) connected to tygon®-tubes (E 3603, inner d: 1.6 mm, Saint - Gobain Performance Plastics) were inserted in the stoppers under sterile conditions. The produced gas was released continuously into 50 mL serum bottles, half filled with autoclave-sterilized saturated solution of KCl serving as a water lock.

### Microbial strains, media and cultivation

The *Methanobacterium formicicum* MF (DSM 1535) and *Methanosarcina barkeri* MS (DSM 800) strains, originally purchased from the German Collection of Microorganisms and Cell cultures (DSMZ), were provided by the working group microbiology of anaerobic systems at the department of Environmental Microbiology of the Helmholtz Centre for Environmental Research, Leipzig, Germany. The *Methanothrix soehngenii* GP6 (DSM 3671) strain was kindly provided by the microbiology laboratory at the department for agrotechnology and food sciences of Wageningen University and Research, Wageningen, The Netherlands. All three methanogens were cultured in the simplified methanogen medium BFS01 containing per liter: 0.348 g K_2_HPO_4_, 0.227 g KH_2_PO_4_, 0.5 g NH_4_Cl, 0.406 g MgCl_2_ × 6 H_2_O, 0.25 g CaCl_2_ × 2 H_2_O, 2.25 g NaCl, 1.42 mg FeCl_2_ × 4H_2_O, 0.85 g NaHCO_3_, 0.3 g L-Cysteine hydrochloride monohydrate, 1 mL trace element solution SL-10, and 1 mL Wolin’s vitamin solution-10 ^71^. Before each inoculation, the BFS01 medium was supplemented with carbon sources and electron donors as indicated in Table 1. All three methanogens were cultured separately in 200 mL serum bottles containing 120 mL of each fully supplemented BFS 01 medium (see Table 1) and 10 mL (~ 7.7% v/v) methanogenic inoculum in the late logarithmic phase and/or early stationary phase. *M. barkeri* and *M. formicicum* were cultured for 3 weeks and *M. soehngenii* for 4 weeks due to its slow growth rate^61,64^. Nearly stable CH_4_ concentration in the culture headspaces over several days was used as an indicator of the maximum cell density achieved for each methanogenic culture. Due to their cell morphology: 1) *M. barkeri*, which aggregated as irregular-sized, interconnected cell clumps^71^, 2) *M. formicicum*, which formed cell colony resembling a sponge-like structure^71^, and 3) *M. soehngenii*, which had a rod-shaped filament morphology^71^, optical density measurement (using OD_600_) and cell counting (using Multisitzer 3 Coulter Counter, Beckmann Coulter TM) were attempted, but regarded as inappropriate. 5 mL of each methanogenic culture were used for process monitoring and the remaining 125 mL were used for the electrochemical experiments, to ensure comparable methanogenic biomass in the replicates of each single experiment (e.g., *M. barkeri* + *Geobacter* spp. biofilm). See specifications and additional information on the BFS01 medium used, growth monitoring and physiological parameters for each methanogen in Dzofou et al. ^71^.

**Table 1:** Growth conditions for methanogenic cultures

	Substrate	Gas phase	pH	Incubation time (weeks)
*M. barkeri*	185 mmol L^−^^1^ methanol, 10 mmol L^−^^1^ acetate	N_2_:H_2_ (97:3, v/v)	7.1	3
*M. formicicum*	50 mmol L^−^^1^ formate, H_2_:CO_2_ (50:50, v/v)	H_2_:CO_2_ (50:50, v/v)	7.5	3
*M. soehngenii*	40 mmol L^−^^1^ acetate	N_2_:CO_2_ (50:50, v/v)	7.6	4

Table 1 summarizes the different substrates, gas phase composition, pH, and incubation time during the growth of the different methanogens.

*Geobacter* spp. biofilms were initially grown according to Gimkiewicz et al. ^73^ using wastewater from a primary clarifier of a local wastewater treatment plant (AZV Parthe, 04551 Borsdorf, Germany). Biofilms were subsequently enriched using a simple electrochemical enrichment procedure according to Liu et al. ^74^. The growth medium used during biofilm enrichment and growth consisted of 50 mmol L^−^^1^ phosphate buffer, amended with 10 mmol L^−1^ sodium acetate, 12.5 mL L^−1^ vitamins and 12.5 mL L^−1^ trace elements^73,75^ (for simplicity referred to as acetate-based medium). For inoculation of a fresh graphite anode, the biofilms were scraped off the anode with a sterile spatula and resuspended in fresh growth medium by vortexing. Several repetitions of this electrochemical enrichment procedure were performed until the turbidity of the planktonic phase after each batch cycle decreased significantly and a strong reddish biofilm was obtained, showing that biofilms dominated by *Geobacter* spp. were received^73,75,76^.

For electrochemical experiments, previously enriched *Geobacter* spp. biofilms were scraped off the anode using a sterile spatula and resuspended in autoclave-sterilized acetate-based medium. Subsequently, the inoculum was used in the MEC to grow new biofilm anodes under strict anoxic conditions.

The MEC were sparged with sterile N_2_ gas (Nitrogen 5.0, Linde AG) for at least 30 min to maintain anoxic conditions prior to each batch cycle and operated at 38 °C using an incubator hood (Unihood 650, UniEquip). To maintain homogeneity and reduce mass transfer limitations, the media were stirred using a magnetic stirrer (Variomag Poly 15, Thermo Scientific) at 250 rpm. All MEC were connected to a multipotentiostat (PARSTAT MC, AMETEK Inc.) for biofilm growth and for the experiments.

Biofilm formation and maturation was performed in MEC using consecutive and repeated cycles of chronoamperometry (CA) for ~23 h at 0.2 V. CA was followed by three cycles of cyclic voltammetry (CV) with vertex potentials at −0.5 V and 0.3 V and a scan rate of 1 mV s^−^^1^. *Geobacter* spp. biofilms were pre-grown over three successive batch cycles with one batch cycle lasting always one week. The nearly constant maximum current density (*j*_max_, normalized maximum current point in each batch cycle to the projected surface area of the working electrode) measured during the batch cycles before exposure of *Geobacter* spp. biofilms to the methanogens, was used as an indicator of biofilm maturation or steady-state.

### Experiments

**Fig. 4:**
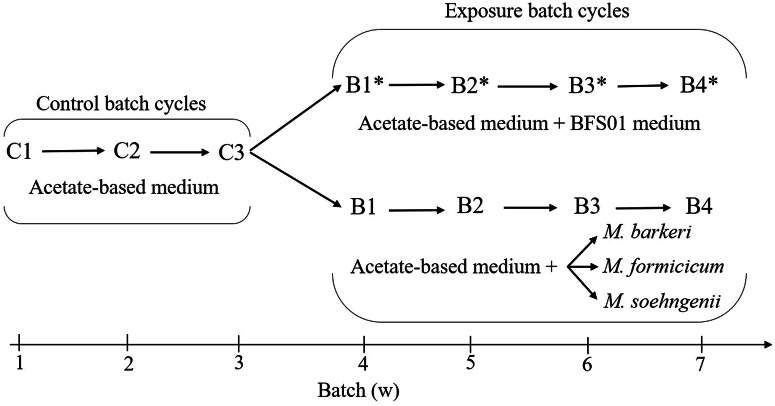
Overview of electrochemical experiments. Each batch cycle always lasts one week. “C1 to C3” indicate the three successive control batch cycles during the pre-growth of *Geobacter* spp. biofilms with 100% acetate-based medium, “Exposure batch cycles” indicate the successive batch cycles during the electrochemical control experiment without methanogens, i.e., B1*-B4* with the 50:50, v/v mixture of acetate-based medium and abiotic BFS01 medium (top) as well as the electrochemical experiment with methanogens, i.e., B1-B4 with the 50:50, v/v mixture of acetate-based medium and each methanogenic culture (bottom).

To study the interactions between *Geobacter* spp. biofilms and the three methanogens, five sets of experiments (four electrochemical and one biological) including two control experiments were conducted (Supplementary Table 1). Figure 4 gives an overview of the electrochemical experiments that were always conducted in batch mode with one batch cycle lasting one week. *Geobacter* spp. biofilms were initially pre-grown in acetate-based medium over three successive batch cycles denoted as control batches C1, C2, and C3. 0.5 mmol L^−^^1^ of the autoclave-sterilized methanogen inhibitor, 2-bromoethanesulfonate (2-BES, Sigma-Aldrich) was added to the acetate-based medium during C1 and C2 to promote anode-respiring bacteria and limit methanogenesis from acetate^77^. During C3, *Geobacter* spp. biofilm was depleted in remaining 2-BES by using only acetate-based medium.

To verify that there was no inhibition of the three methanogens during the electrochemical experiments described below, a biological control experiment was performed to test the activity of *M. barkeri, M. soehngenii*, and *M. formicicum* in the media used to grow *Geobacter* spp. biofilms, i.e., acetate-based medium. In case of *M. formicicum*, acetate was replaced by 50 mmol L^-1^ formate. 200 mL serum bottles containing 120 mL of acetate-based medium were inoculated with 10 mL (~ 7.7% v/v inoculum) of actively growing *M. barkeri* and *M. soehngenii* cultures and monitored for 42 days. A similar setup containing 120 mL of formate-based medium (i.e., 50 mmol L^−^^1^ phosphate buffer, supplemented with 50 mmol L^−^^1^ formate, 12.5 mL L^−^^1^ vitamins, and 12.5 mL L^-1^ trace elements) was inoculated with 10 mL (~ 7.7% v/v inoculum) of actively growing *M. formicicum* culture and monitored for 42 days. The gas phase compositions in each control were identical to those in Table 1. The gas phase in the *M. formicicum* culture was renewed once a week to avoid underpressure due to H_2_ and CO_2_ consumption for CH_4_ production.

An electrochemical control experiment was performed to ensure that *Geobacter* spp. biofilms were not inhibited by the BFS01 medium and to subsequently compare methanogen-induced changes in the electrochemical activity, stability, and microbial community of *Geobacter* spp. During the electrochemical control experiment, the activity of pre-grown *Geobacter* spp. biofilms was monitored over four successive exposure batch cycles, denoted B1*, B2*, B3*, and B4*, by immersing biofilms in a 50:50, v/v mixture of acetate-based medium and abiotic anaerobic BFS01 medium.

To study the specific interactions between *Geobacter* spp. biofilms and each methanogen, three electrochemical experiments were performed. Pre-grown *Geobacter* spp. biofilms were immersed in anaerobic and freshly prepared 50:50, v/v mixtures of acetate-based medium and each methanogenic culture grown in the BFS01 medium (see Supplementary Table 1). Interactions between *Geobacter* spp. biofilms and methanogens were monitored over four successive exposure batch cycles, denoted B1, B2, B3, and B4. Prior to each experiment, the remaining acetate concentration in the methanogenic cultures (except those of *M. formicicum*) was measured one day in advance as previously described^32^. The final acetate concentration in the 50:50, v/v mixtures was subsequently adjusted to ~10 mmol L^−^^1^.

### Chemical and electrochemical analysis

The growth of each methanogenic culture was monitored weekly using chemical oxygen demand removal (ΔCOD), consumption of acetate and formate measured using high performance liquid chromatography (HPLC), and headspace gas composition measured using a gas chromatograph (GC).

ΔCOD was measured using COD cuvette tests (LCK 014, Hach-Lange), according to the manufacturer’s instructions. The COD removal efficiency was calculated as follows:

1 $$\%\, {COD}=\frac{{{COD}}_{{t}_{0}}-{{COD}}_{t}}{{{COD}}_{{t}_{0}}}\times 100$$

Where ${{COD}}_{{t}_{0}}$ is the initial ${COD}$ concentration at *t*_*o*_ and ${COD}$_*t*_ the ${COD}$ concentration at the sampling point *t*^78^.

For measuring the acetate and formate concentrations in the methanogenic cultures and the acetate concentration in the MEC over the experiments, 1 mL aliquots were sampled from the serum bottles and the MEC at inoculation and then once a week. The samples were filtered using 0.2 µm syringe filters (Nylon, VWR) and stored at −20 °C or analysed immediately using HPLC (Shimadzu Scientific Instruments) equipped with a refractive index detector RID 10 A, a prominence diode array detector SPD.M20A, and a HiPlex H column (300 × 7.7 mm, 8 mm pore size, Agilent Technolgies) with a pre-column HiPlex H column (50 mm × 7.7 mm, 8 mm pore size, Agilent Technolgies). The sample volume for HPLC measurement was 200 µL and the injection volume was 20 µL. 5 mmol L^−^^1^ sulfuric acid was used as isocratic mobile phase with a flow rate of 0.7 mL min^−^^1^ at 60 °C, over a total run time of 60 min.

CH_4_, H_2_, and CO_2_ concentrations in the headspace of each serum bottle and MEC were determined weekly. Therefore, two replicates of 1 mL gas samples were taken from the headspace of each serum bottle and MEC using sterile needle-syringe arrangements rendered anoxic by flushing with sterile oxygen-free N_2_ gas. Each gas sample was injected into glass vials pre-flushed for 30 min with argon (Argon 4.8, Linde AG). Gas composition was analyzed using a GC equipped with an autosampler (Perkin Elmer Inc, Waltham). The GC was equipped with HayeSep N/Mole Sieve 13X columns and a thermal conductivity detector. The oven and detector temperatures were 60 °C and 200 °C, respectively. The carrier gas was argon. Each gas sample was analysed within 4 h after sampling.

The electrochemical activity of *Geobacter* spp. biofilms during electrochemical experiments was measured by consecutive and repeated CA and CV cycles, as during biofilm formation and maturation. CA data were analyzed for 1) maximum current density (*j*_max_)^32,79^, 2) total transferred amount of charge ($$Q)$$^5,32^, 3) ΔCOD^32^ and 4) coulombic efficiency (*CE, percentage of the electrons present in the substrate acetate that is recovered as current*)^79,80^. The *CE* during the electrochemical experiments was determined for each batch cycle using Eq. 2.

2 $${CE}=\frac{{M}_{{Ac}}\int {idt}}{{zFV}\Delta c}\times 100$$

M_Ac_ = 59.04 g mol^−^^1^ is the molar mass of acetate, V = 250 mL is the medium volume in the MEC, F is the Faraday constant (F = 96,485.34 C mol^−^^1^), z = 8 is the number of released electrons during oxidation of acetate, Δc = c_0_-c_1_ is the difference in acetate concentration that is acetate consumption in g L^−^^1^ measured by HPLC and $\int {idt}$ is the total transferred amount of charge (*Q*), calculated by integrating the current over time^73^.

CV measurements were only performed for quality control (no data is reported). Furthermore, each electrochemical experiment was monitored weekly by measuring pH (pH 3310, WTW) and conductivity of the media (Cond 3110, WTW).

### Microbial community analysis

Samples for microbial community analysis were taken at the end of each electrochemical experiment from the biofilm anodes and from the planktonic phase of each replicate. Biofilm samples were scratched off from the anodes using a sterile spatula. Both, planktonic and biofilm samples were centrifuged at 10,000 × g for 10 min and the pellets were stored in sterile 2 mL microcentrifugation tubes at −20 °C.

Genomic DNA was extracted from biofilm samples and selected planktonic samples using the Quick-DNA™ Fecal/Soil Microbe Miniprep Kit (ZYMO RESEARCH). DNA concentrations were measured by fluorescence quantification using the Qubit^TM^ dsDNA BR Assay Kit (Thermo Fisher Scientific) and a Qubit^TM^ 4 Fluorometer (Thermo Fisher Scientific). The purity of the DNA was evaluated by microvolume absorbance on a DS-11 FX+ (Denovix), where A260/A280 and A260/A230 values around 1.8 and 2.0, respectively, are considered as pure nucleic acid.

The bacterial and archaeal diversity of the samples was determined by metabarcode sequencing, as previously described^71^.

Since metabarcoding only covers the V3-V4 region of the 16S rRNA gene, which does not allow for good differentiation down to the species level, but is limited to the genus or in some cases only the family level, MiSeq whole metagenome shotgun sequencing of biofilm samples was performed and the relative read-mapping results compared with the MiSeq amplicon sequencing results. Sequencing libraries were prepared according to the Nextera XT DNA Library Prep Kit (Illumina) and sequenced on an Illumina MiSeq with the MiSeq Reagent Kit v3 in a paired-end mode and read lengths of 300 bp. Raw sequencing data was demultiplexed and converted to fastq-files in Local Run manager (Illumina). The data were further processed in the CLC Genomics Workbench 22.0.1 (Qiagen). The raw reads were filtered using Trim Reads 2.6, and mapped against the 5 reference genomes (*G. anodireducens*, RefSeq GCF_014883105.1; *G. sulfurreducens*, RefSeq GCF_019904315.1; *M. soehngenii*, RefSeq GCF_000204415.1; *M. formicicum*, RefSeq GCF_000762265.1, and *M. barkeri* RefSeq GCF_000970025.1) using Map Reads to Reference 1.8 with default parameters. The archaea reference genomes were previously found to be suitable references for these 3 species^71^.

To shed more light onto the biofilm composition, metagenome nanopore sequencing of biofilm samples was performed and the results compared to the metabarcoding and MiSeq shotgun sequencing results. Sequencing libraries were generated using the ligation sequencing gDNA - native barcoding Kit (Oxford Nanopore Technologies, SQK-NBD112.24). DNA libraries were sequenced using a MinION instrument equipped with a FLO-MIN112 Flow Cell (Oxford Nanopore Technologies). The MinKNOW version 22.03.5 (Oxford Nanopore Technologies) was used for sequencing and basecalling was performed separately with guppy version 6.0.6 (Oxford Nanopore Technologies). Raw nanopore sequence data were base-called using Guppy version 6.1.3 (Oxford Nanopore Technologies) with the command “guppy_basecaller -i fast5 -s fastq -c dna_r10.4_e8.1_sup.cfg -x ‘cuda:0’ --trim_adapters --trim_primers --trim_barcodes --compress_fastq --min_qscore 10 --barcode-kits SQK-NBD112-24”. Basecalled reads were then filtered using Nanofilt (version 2.8.0)^81^ with a minimum length of 1000 bp and a minimum PHRED score of Q10, followed by porechop (version 0.2.4) to ensure removal of adapter sequences. Then, Kraken2^82^ (version 2.1.2) and Bracken (version 2.7) were run for each sample using 1) a custom database created from the same 5 reference genomes used for the shotgun read-mapping (see above) and 2) a Kraken standard database with date-stamp June 7, 2022, downloaded from https://benlangmead.github.io/aws-indexes/k2. These analyses were automated using snakemake (version 7.7.0), see supplementary snakemake files. Finally, the Kraken and Bracken results were analyzed using Pavian^83^ (https://fbreitwieser.shinyapps.io/pavian).

### Reporting summary

Further information on research design is available in the Nature Research Reporting Summary linked to this article.

### Supplementary information

Supplementary Information

Reporting Summary

## Data Availability

The raw sequencing reads have been submitted to the Sequence Read Archive (https://www.ncbi.nlm.nih.gov/sra) and are available under Bioproject PRJNA894981.
