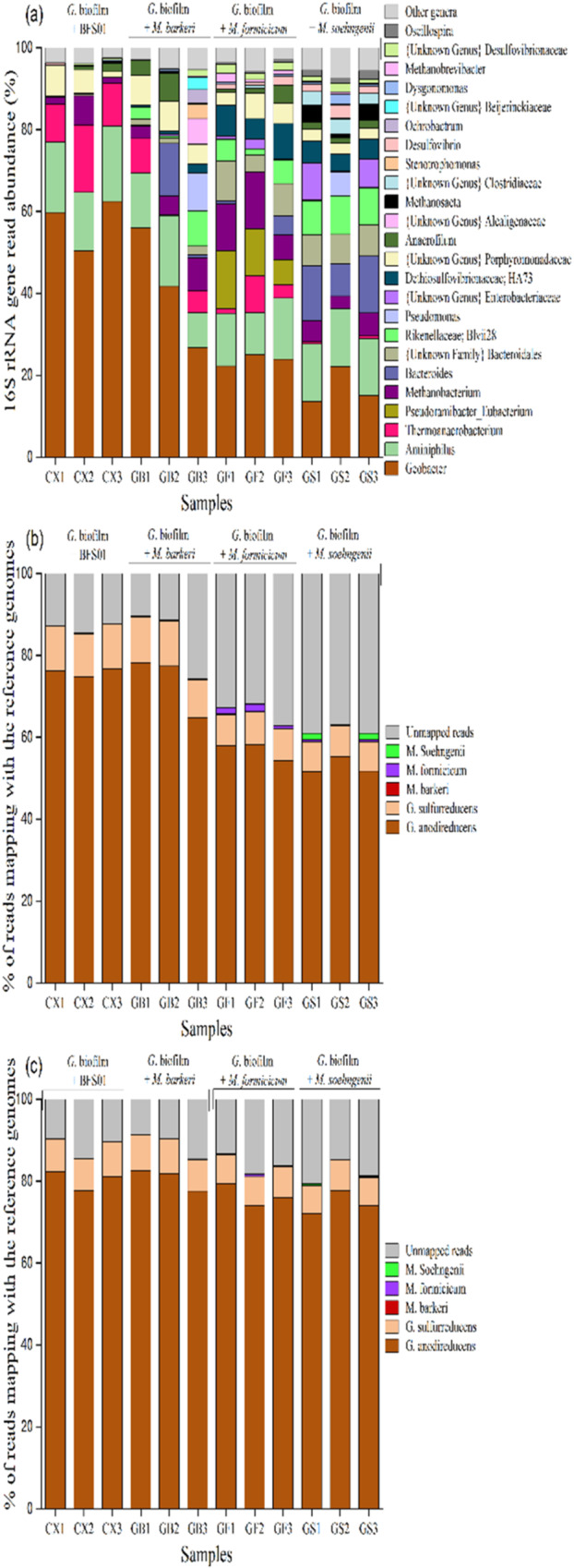# Author Correction: Effect of model methanogens on the electrochemical activity, stability, and microbial community structure of *Geobacter* spp. dominated biofilm anodes

**DOI:** 10.1038/s41522-024-00513-9

**Published:** 2024-04-12

**Authors:** Daniel Dzofou Ngoumelah, Tonje Marita Bjerkan Heggeset, Tone Haugen, Snorre Sulheim, Alexander Wentzel, Falk Harnisch, Jörg Kretzschmar

**Affiliations:** 1 DBFZ Deutsches Biomasseforschungszentrum gemeinnützige GmbH (German Biomass Research Centre), Department of Biochemical Conversion, 04347 Leipzig, Germany; 2 SINTEF Industry, Department of Biotechnology and Nanomedicine, 7034 Trondheim, Norway; 3 Helmholtz Centre for Environmental Research - UFZ, Department of Microbial Biotechnology, 04318 Leipzig, Germany; 4 Zittau/Görlitz University of Applied Sciences, Faculty of Natural and Environmental Sciences, 02763 Zittau, Germany

**Keywords:** Applied microbiology, Next-generation sequencing

Correction to: *npj Biofilms and Microbiomes* 10.1038/s41522-024-00490-z, published online 05 March 2024

In this article the wrong figure appeared as Fig. 3; the figure should have appeared as shown below. The original article has been corrected.