# Supplementary material for: Effect of model methanogens on the electrochemical activity, stability, and microbial community structure of Geobacter spp. dominated biofilm anodes
Source: NPJ Biofilms Microbiomes. 2024 Mar 5;10:17. doi: 10.1038/s41522-024-00490-z (PMC10915144; doi:10.1038/s41522-024-00490-z)
Supplement: Supplementary file 1 — Supplementary Information [file 41522_2024_490_MOESM1_ESM.pdf]

Supplementary Information

Effect of model methanogens on the electrochemical activity, stability, and microbial community structure of *Geobacter* spp. dominated biofilm anodes

*Daniel Dzofou Ngoumelah[1], Tonje Marita Bjerkan Heggeset[3], Tone Haugen[3], Snorre Sulheim[3], Alexander Wentzel[3], Falk Harnisch[2], Jörg Kretzschmar[1,4,]**

[1]DBFZ Deutsches Biomasseforschungszentrum gemeinnützige GmbH (German Biomass Research Centre), Department of Biochemical Conversion, 04347 Leipzig, Germany

[2]Helmholtz Centre for Environmental Research - UFZ, Department Microbial Biotechnology, 04318 Leipzig, Germany

[3]SINTEF Industry, Department of Biotechnology and Nanomedicine, 7034 Trondheim, Norway

[4]University of Applied Sciences Zittau/Görlitz, Faculty of Natural and Environmental Sciences, 02763 Zittau, Germany

Table of content

1 SUPPLEMENTARY METHODS 2

2 SUPPLEMENTARY RESULTS 2

3 SUPPLEMENTARY REFERENCES 10

1 SUPPLEMENTARY METHODS

Supplementary Table 1. Parameters of the performed experiments, t: duration of the experiment, n: number of biological replicates per experiment.

Name of the experiment	Age of methanogens (weeks)	Age of *Geobacter* spp. biofilms (weeks)	Mixture		n	t (days)
			Acetate/formate-based medium (%)	Methanogenic cultures (%)		
Biological Control	-	-	92.3	7.7	1	42
Electrochemical Control	-	3	50	50*	4	49
M. barkeri + *Geobacter* spp. biofilms	3	3	50	50	3	49
M. formicicum + *Geobacter* spp. biofilms	3	3	50	50	4	49
M. soehngenii + *Geobacter* spp. biofilms	4	3	50	50	4	49

*BFS01 medium (methanogen-free growth medium)

2 SUPPLEMENTARY RESULTS

- **Activity of methanogens in *Geobacter* spp. media**

During the biological control experiment, the activities of *M. barkeri* and *M. soehngenii* were monitored in acetate-based medium and that of *M. formicicum* in formate-based medium (Supplementary Fig. 1). Acetate consumption by *M. barkeri* and *M. soehngenii* occurred at a slow rate, in contrast to *M. formicicum*, which depleted formate within 42 days (Supplementary Fig. 1a). Headspace analysis of *M. formicicum* from day 7 to day 42, indicated a CH_4 concentration ~35 % versus CH_4 concentrations <10 % for *M. barkeri* and *M. soehngenii* (Supplementary Fig. 1b).

M. barkeri is known as a mixotrophic methanogen with a high metabolic affinity for methanol and a low affinity for acetate[1–3]. The omission of methanol in the *M. barkeri* culture and the use of an acetate-based medium that is not adapted for methanogens and with a different composition than the BFS01 medium, may explain why the CH_4 concentration in the culture headspace did not reach the log phase during 42 days of incubation. *M. soehngenii* is known as a strict acetoclastic methanogen, with a lag phase of several days and slow growth rate even for optimal conditions[1,4,5]. This is consistent with the low CH_4 concentration measured

in the culture headspace during the whole incubation period. Furthermore, the different chemical composition of the acetate-based medium compared to BFS01 may also explain the slow growth or low CH_4 concentration in the *M. soehngenii* culture. *M. formicicum* is known as a strict hydrogenotrophic methanogen using H_2-CO_2 or formate as substrate, with short lag phase and a log phase peaking rapidly[6–8]. The stationary phase of *M. formicicum* grown in formate-based medium was reached after ~7 days of incubation (Supplementary Fig. 1b), and is similar to that of previous work using the BFS01 medium[9].

The acetate and formate consumption profiles, as well as the CH_4 production, shown in Supplementary Fig. 1, indicate that *M. barkeri* and *M. soehngenii* remained active in the acetate-based medium, as well as *M. formicicum* in the formate-based medium, albeit at different activity levels. Here, we only proved activity but not growth of the three methanogens in the combination of BFS01 medium and acetate/formate-based medium. Therefore, it was concluded that mixing 50:50, v/v of acetate-based medium with pre-grown methanogenic cultures in BFS01 medium for studying the different interactions with *Geobacter* spp. biofilms in the MEC setup is not detrimental to the methanogens.

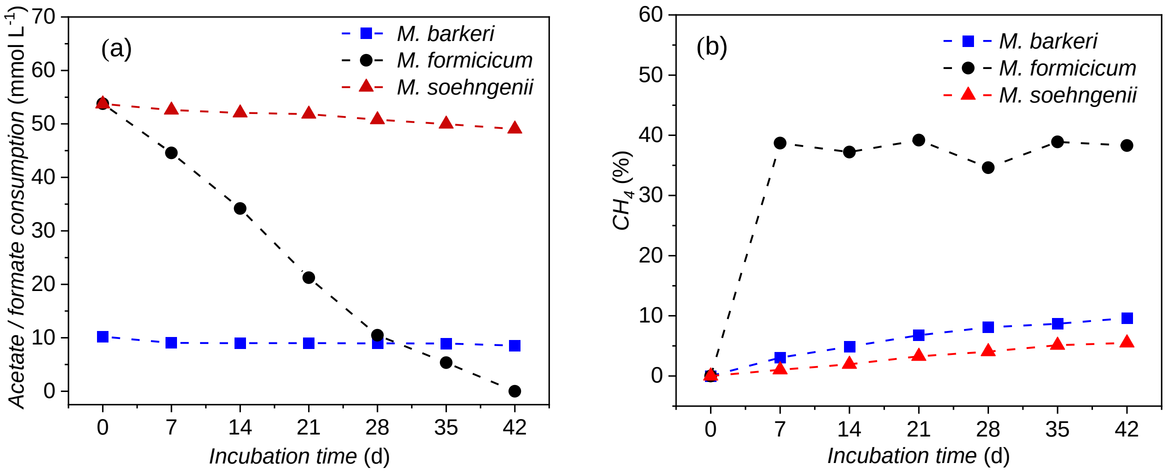

Supplementary Figure 1. Process parameters during incubation of *M. barkeri*, and *M. soehngenii* in acetate-based medium and *M. formicicum* in formate-based medium.

(a) acetate (blue and red dots) and formate (black dots) consumption, (b) CH_4 concentration in the headspace of the control bottles, n = 1.

Supplementary Table 2. Coulombic efficiency (CE) calculated at the end of each batch cycle during the electrochemical experiments (see Fig. 4). For the age of each methanogenic culture, please see Table 1 in the main text, n ≥ 3, errors indicate confidence interval CI.

Experiment	Batches	CE (%)
Electrochemical control	C1	83.90±5.94
	C2	78.38±18.04
	C3	83.07±16.85
	B1*	75.55±16.97
	B2*	77.52±9.01
	B3*	83.68±20.29
	B4*	75.76±21.33
M. barkeri + *Geobacter* spp. biofilms	C1	85.01±2.58
	C2	101.97±6.74
	C3	119.33±14.79
	B1	109.90±20.26
	B2	142.95±48.87
	B3	149.82±12.86
	B4	125.09±5.60
M. barkeri + *Geobacter* spp. biofilms$	C1	90.52±1.22
	C2	81.99±5.90
	C3	74.51±3.76
	B1	165.48±36.37
	B2	143.09±5.86
	B3	132.08±23.18
	B4	120.70±6.99
M. formicicum + *Geobacter* spp. biofilms	C1	80.04±3.21
	C2	79.46±5.97
	C3	91.11±17.04
	B1	96.41±5.01
	B2	104.69±4.61
	B3	95.05±45.04
	B4	105.66±4.73
M. soehngenii + *Geobacter* spp. biofilms	C1	69.57±7.10
	C2	72.87±5.11
	C3	70.55±3.37
	B1	46.72±5.45
	B2	49.72±5.07
	B3	33.79±4.88
	B4	44.55±6.22

$indicates exposure of *Geobacter* spp. biofilms to *M. barkeri* aged 3-6 weeks

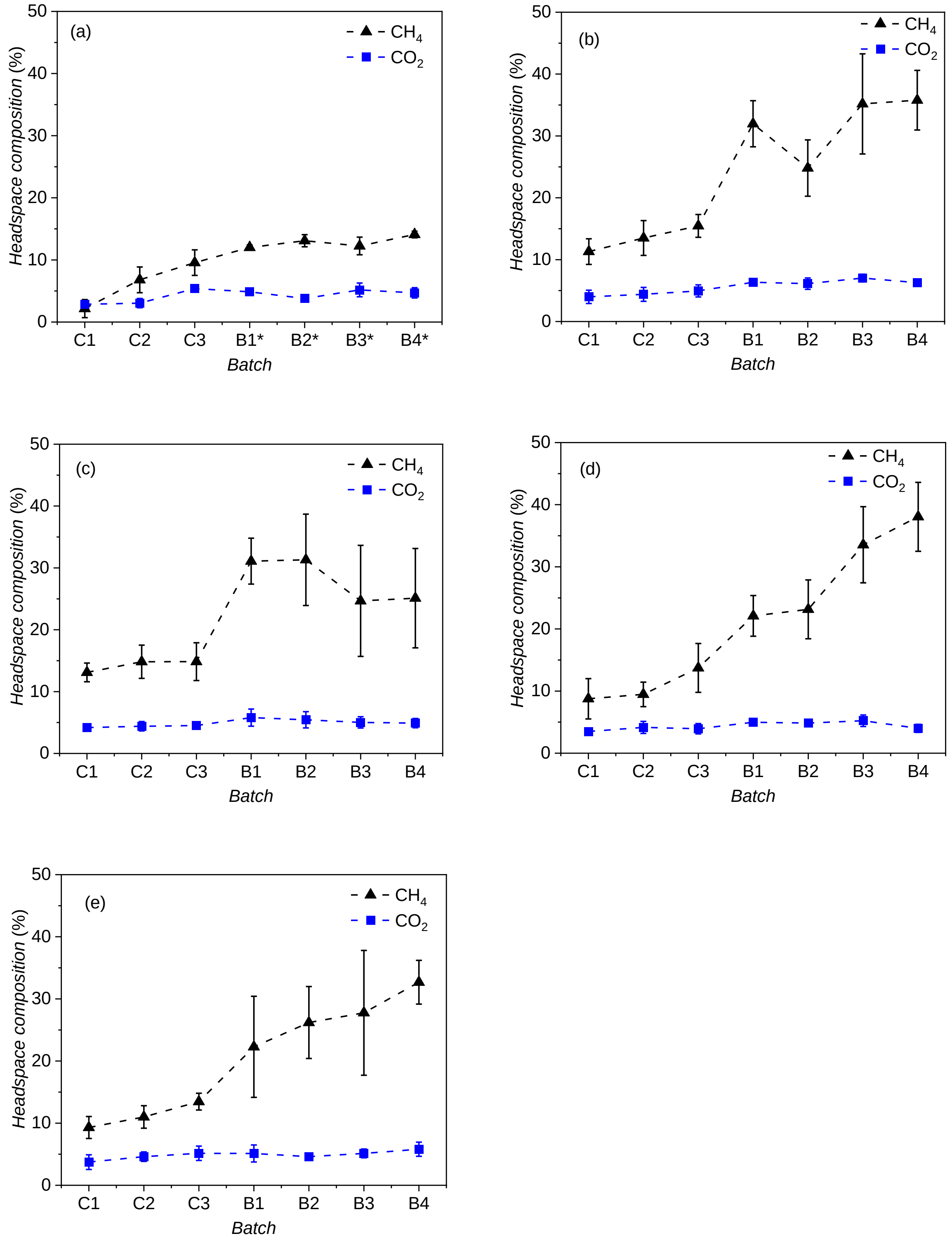

Supplementary Figure 2. MEC headspace composition. CH_4 and CO_2 concentration in the headspace of the MEC at the end of each batch cycle upon exposure of *Geobacter* spp. biofilm to a 50:50, v/v mixture of acetate-based medium and: (a) abiotic BFS01 medium, (b) *M. barkeri* cultures aged 3 weeks in BFS01, (c) *M. formicicum* cultures aged 3 weeks in

BFS01, (d) *M. soehngenii* cultures aged 4 weeks in BFS01, (e) *M. barkeri* cultures aged 3-6 weeks in BFS01. C1-C3: control batches with only acetate-based medium, B1*-B4*: four successive batch cycles with abiotic BFS01 medium, B1-B4: four successive batch cycles with the methanogenic cultures, $n \geq 3$, error bars indicate confidence interval CI.

The methane formation in the headspace of the MEC was monitored over each experiment (Supplementary Fig. 2). In general, the average methane concentration in the MEC of all experiments increased slightly but insignificantly during the control batches, showing a value ≤ 15 % in C3. The latter observation indicates that the concentration of 0.5 mmol L^{-1} 2-bromoethanesulfonate (2-BES) that was used in C1 and C2 did not entirely inhibit methanogenesis from acetate. The methane concentration in the MEC remained nearly constant upon exposure of *Geobacter* spp. biofilms to the 50:50, v/v mixture of acetate-based medium and abiotic BFS01 medium (i.e., from B1* to B4*) and did not increase significantly compared to the last control batch cycle C3 (Supplementary Fig. 2a). In contrast, compared to the last control batches, the methane concentration in the MEC increased significantly upon exposure of *Geobacter* spp. biofilms to each methanogen (Supplementary Fig. 2b, 2c, 2d, and 2e). The latter observation is further evidence for the continuing activity of the three methanogens in the MEC, even after dilution to 50:50, v/v with acetate-based medium.

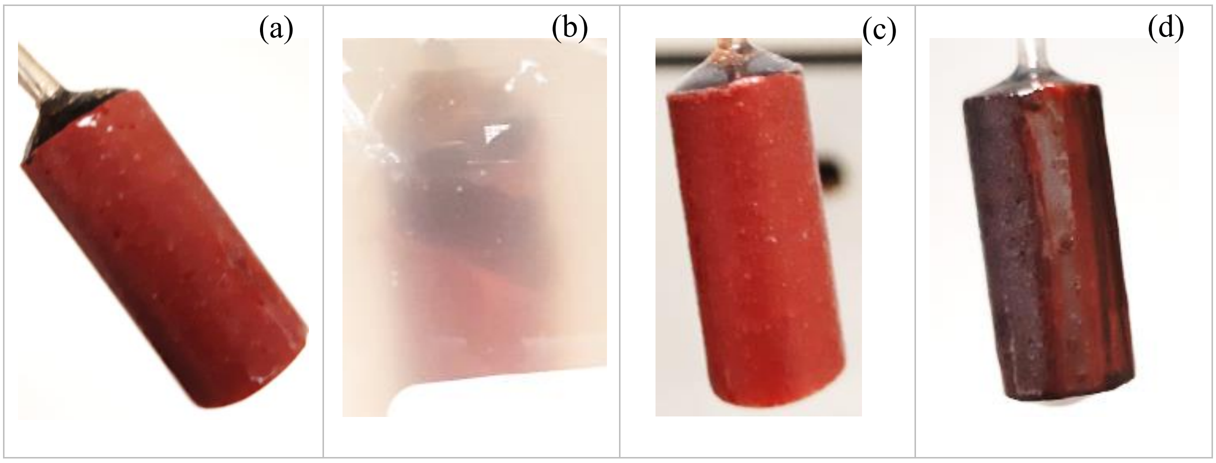

Supplementary Figure 3. Photographs of *Geobacter* spp. dominated biofilms on a graphite rod electrode under varied exposures. (a) at the end of the 4th exposure batch (B4*) to a 50:50, v/v mixture of acetate-based medium and BFS01 medium, (b) during the 2nd exposure batch (B2) to a 50:50, v/v mixture of acetate-based medium and *M. barkeri* culture in Fig. 1b, (c) at the end of the 4th exposure batch (B4) to a 50:50, v/v mixture of acetate-based medium and *M. formicicum* culture, (d) at the end of the 4th exposure batch (B4) to a 50:50, v/v mixture of acetate-based medium and *M. soehngenii* culture (the biofilm was scraped on one side to assess the changes in staining between the outer and inner layers of the biofilm).

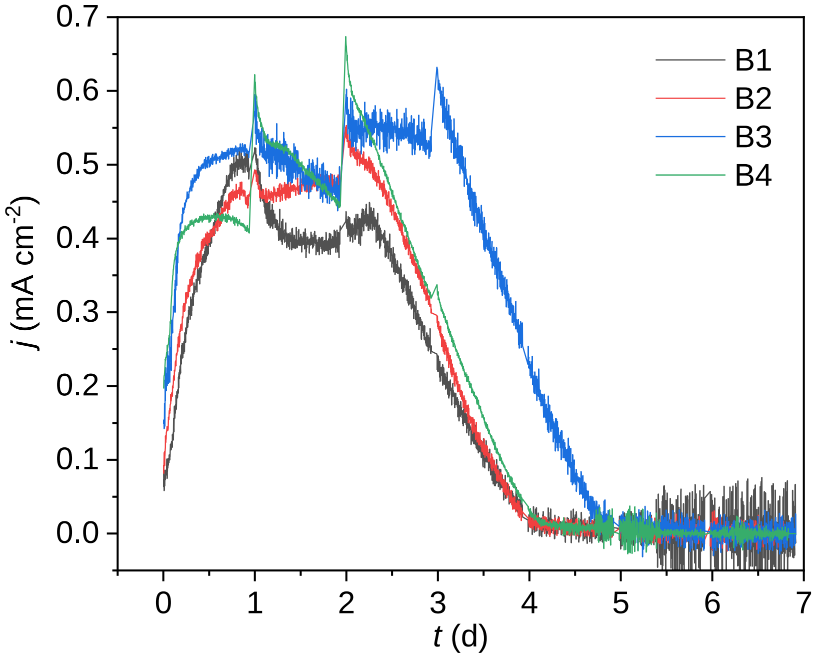

Supplementary Figure 4. Selected chronoamperograms upon exposure of *Geobacter* spp. biofilm to *M. soehngenii* culture. Data from channel 2 out of 4 replicates. B1-B4: four successive batch cycles with *Geobacter* spp. biofilms exposed to *M. soehngenii* cultures.

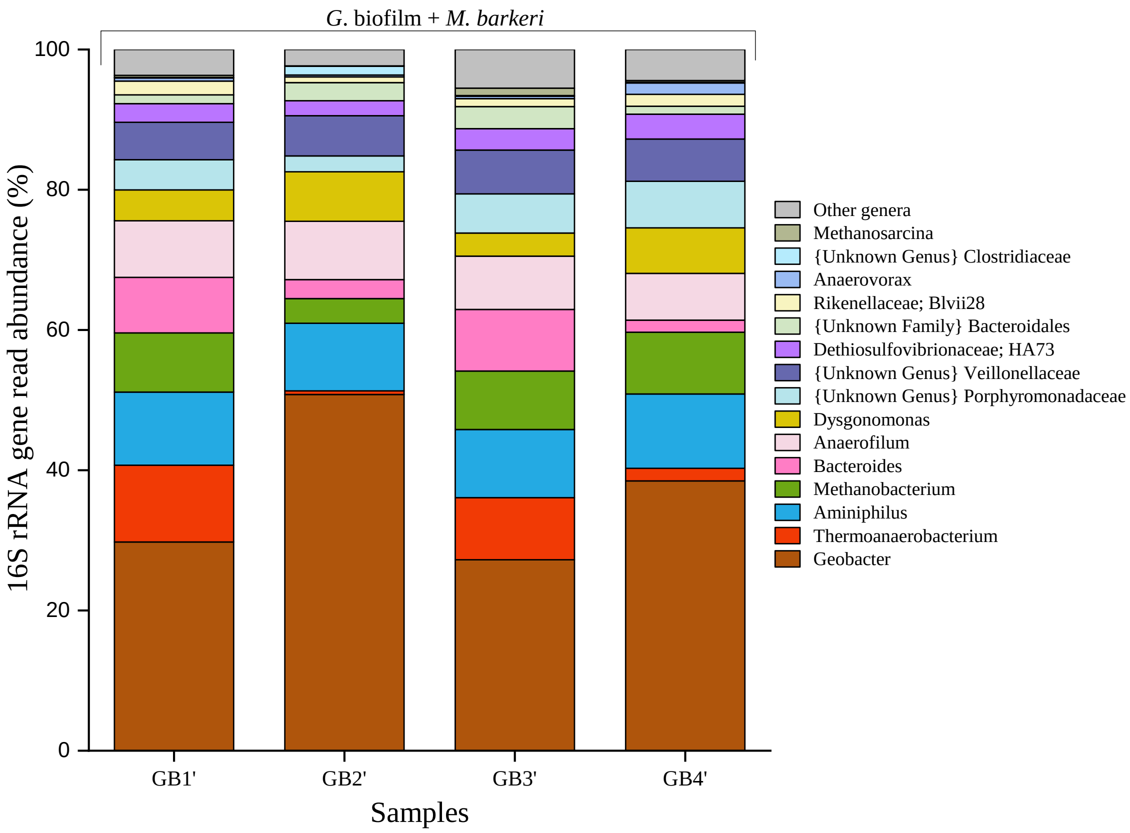

Supplementary Figure 5. Characterization of biofilm samples. Metabarcoding abundance profiling targeting the 16S rRNA V3-V4 region of biofilm samples at the end of the experiment upon exposure of *Geobacter* spp. biofilm to *M. barkeri* aged 3 to 6 weeks from B1 to B4 (also see Fig. 2). [GB1'-GB4'] indicate four replicate biological samples from biofilm exposure to *M. barkeri*. (Other genera < 1 %)

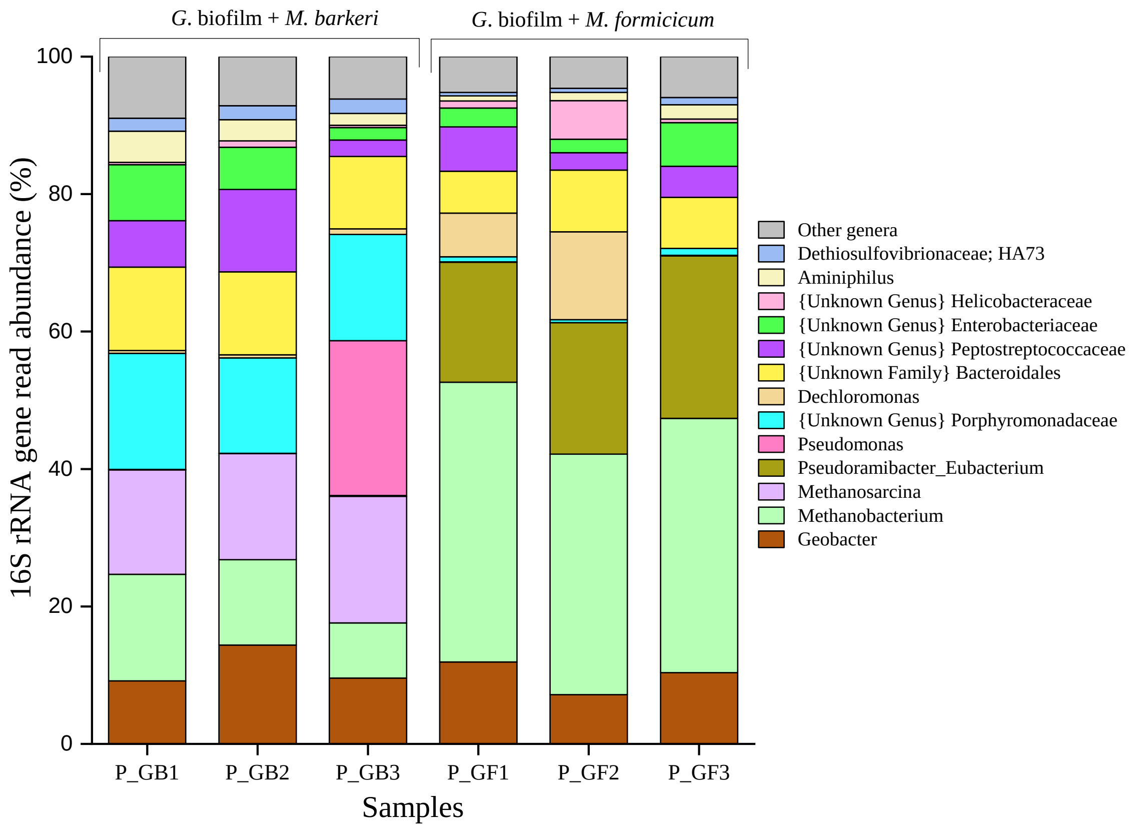

Supplementary Figure 6. Characterization of planktonic samples. Metabarcoding abundance profiling targeting the 16S rRNA V3-V4 region of the planktonic phase in B4 after exposure of *Geobacter* spp. biofilms to *M. barkeri* [P_GB1- P_GB3] and *M. formicicum* [P_GF1- P_GF3], aged three weeks, respectively. (Other genera < 2 %).

3 SUPPLEMENTARY REFERENCES

1. Jetten, M. S. M., Stams, A. J. M. & Zehnder, A. J. B. Methanogenesis from acetate: a comparison of the acetate metabolism in Methanothrix soehngenii and Methanosarcina spp. *FEMS Microbiol. Rev.* **88,** 181–198; 10.1007/0-387-30743-5_9 (1992).

2. Jetten, M. S. M., Stams, A. J. M. & Zehnder, A. J. B. Isolation and Characterization of Acetyl-Coenzyme A Synthetase from Methanothrix soehngenii **171,** 5430–5435; 10.1128/jb.171.10.5430-5435.1989 (1989).

3. Bryant, M. P. & Boone, D. R. Emended Description of Strain MST (DSM 800T), the Type Strain of Methanosarcina barkeri. *Int J Syst Bacteriol.* **32,** 169–170; 10.1099/00207713-37-2-169 (1987).

4. Huser, B. A., Wuhrmann, K. & Zehnder, A. J. B. Methanothrix soehngenii gen. nov. sp. nov., a New Acetotrophic Non-hydrogen-oxidizing Methane Bacteriumbacterium. *Arch. Microbiol.* **132,** 1–9; 10.1007/BF00690808 (1982).

5. Touzel, J. P., Prenzier, G., Roustan, J. L., Thomas, I. & Dubourguier, C., Albagnac, G. Description of a New Strain of Methanothrix soehngenii and Rejection of Methanothrix concilii as a Synonym of Methanothrix soehngenii. *Int J Syst Bacteriol.* **38,** 30–36; 10.1099/00207713-38-1-30 (1988).

6. Neil, L. S., David, P. B. & James, G. F. Kinetics of Formate Metabolism in Methanobacterium formicicum and Methanospirillum hungatei. *Appl. Environ. Microbiol.* **44,** 549–554; 10.1128/aem.44.3.549-554.1982 (1982).

7. Bryant, M. P. & Boone, D. R. Isolation and Characterization of Methanobacterium forrnicicum MF. *Int J Syst Bacteriol.* **37,** 171; 10.0000/PMID13549377 (1987).

8. Neil, L. S. & James, G. F. Metabolism of Formate in Methanobacterium formicicum. *J. Bacteriol.* **142,** 800–807; 10.1128/jb.142.3.800-807.1980 (1980).

9. Dzofou Ngoumelah, D. *et al.* A unified and simple medium for growing model methanogens. *Frontiers in microbiology* **13:1046260**; 10.3389/fmicb.2022.1046260 (2023).
